# Bridging the Cartesian Divide Between Organic and Functional Catatonia: A Case Report

**DOI:** 10.7759/cureus.96402

**Published:** 2025-11-09

**Authors:** Haameem Syed, David Shakespeare, Rawan AbouHatab, Matthew Miller

**Affiliations:** 1 Neurology, Lancashire Teaching Hospitals, Preston, GBR; 2 Neurorehabilitation, Lancashire Teaching Hospitals, Preston, GBR; 3 Psychiatry, Lancashire Teaching Hospitals, Preston, GBR

**Keywords:** catatonia, electroconvulsive therapy, functional recovery, intracranial surgery, lithium maintenance therapy, meningioma resection, multidisciplinary management, neurorehabilitation, organic brain injury, postoperative neuropsychiatry

## Abstract

Electroconvulsive therapy (ECT) is a well-established and highly effective treatment for catatonia. Historically, a history of intracranial surgery was regarded as a relative contraindication. This was because of concerns about increased intracranial pressure and postoperative vulnerability. However, emerging evidence supports the safe use of ECT in patients with previous neurosurgical procedures when multidisciplinary team discussion confirms anatomical stability. We describe a woman in her late fifties who developed profound catatonia nearly one year after undergoing left pterional craniotomy and meningioma resection. She presented with mutism, immobility, and refusal to eat or drink. Investigations excluded tumor recurrence, infection, metabolic disturbances, and autoimmune encephalitis. Despite treatment with high-dose lorazepam, titrated up to 2 mg four times daily, her symptoms persisted. Following multidisciplinary review, including neurosurgical confirmation of stable postoperative anatomy, normal intracranial pressure and absence of mass effect, modified bilateral ECT was initiated. The patient tolerated treatment well with no complications and demonstrated gradual, sustained improvement across motor, cognitive, and affective domains after approximately twenty-six sessions. Lithium was subsequently introduced to support mood stabilization and functional recovery, with plans to taper ECT once lithium efficacy was established. She continues to receive ECT as part of a multidisciplinary neurorehabilitation program with improvements in communication and social engagement. This case illustrates that ECT is a viable, safe, and effective intervention for catatonia even in patients with a history of intracranial surgery. It also highlights ECT’s role not only as a psychiatric treatment but as a neuromodulatory therapy capable of restoring motor and volitional function following structural brain injury. The case thereby bridges the traditional divide between *organic* and *functional* paradigms of neuropsychiatric conditions.

## Introduction

Catatonia is a neuropsychiatric syndrome characterized by a constellation of motor, behavioral, and affective abnormalities. The clinical features include mutism, stupor, posturing, waxy flexibility, catalepsy, agitation, and negativism. The use of electroconvulsive therapy (ECT) has increased over the past few decades, with recent studies indicating that ECT need not be reserved solely as a treatment of last resort [1,2]. Its acceptance has grown steadily. Despite its efficacy, the application of ECT in patients with a known history of intracranial pathology has been historically limited because of theoretical concerns regarding increased intracranial pressure and neurological complications [3,4].

Recent literature has re-evaluated these relative contraindications. Evidence from systematic reviews indicates that, with appropriate patient selection and thorough preoperative assessment, ECT can be safely administered to individuals with benign intracranial lesions, such as meningiomas, provided there is no mass effect [3]. Moreover, there is increasing recognition that catatonia may arise from both functional and structural disturbances within shared neural networks. This perspective has shifted the understanding of catatonia from a purely psychiatric syndrome to one that reflects complex network dysfunction across cortical and subcortical regions.

## Case presentation

A 57-year-old woman with no prior psychiatric history presented to the emergency department with a two-week history of global decline, mutism, immobility, poor oral intake, and refusal to engage. There was no history of infection, seizures, or recent trauma. Sixteen months before this admission, she underwent a left pterional craniotomy for resection of an en-plaque meningioma involving the orbital apex and lateral cavernous sinus wall, along with excision of a left occipital sebaceous cyst. The postoperative course was uncomplicated. Her only other past medical history was psoriasis.

At approximately 11 months post-surgery, her family observed progressive behavioural and cognitive changes, including irritability, confusion, disorientation, and bizarre behaviour.

Three months later (14 months post-surgery), she was readmitted to the hospital with severe depressive symptoms and suicidality. During this admission, her presentation evolved, with the emergence of psychotic features, including paranoia, confusion, and disorganised behaviour. She was treated with olanzapine and later switched to aripiprazole due to metabolic side effects. The working diagnosis at that time was psychotic depression.

Two months after that (16 months post-surgery), her condition deteriorated further, with complete mutism, immobility, and refusal to eat or drink, leading to her current admission.

 On examination, she was alert with eyes open but mute, showing marked psychomotor retardation, rigidity, waxy flexibility, and posturing. She intermittently followed simple commands but made no spontaneous movements or verbal responses. Reflexes were brisk, particularly on the left, with a right-sided Hoffmann’s sign. There were no cranial nerve abnormalities. The Bush-Francis Catatonia Rating Scale (BFCRS) score was 8 out of 21, indicating the presence of stupor (2/3), mutism (3/3), and gegenhalten (2/3), along with additional features including catalepsy, waxy flexibility, posturing, and negativism. There were no signs of malignant catatonia or neuroleptic malignant syndrome.

**Figure 1:**
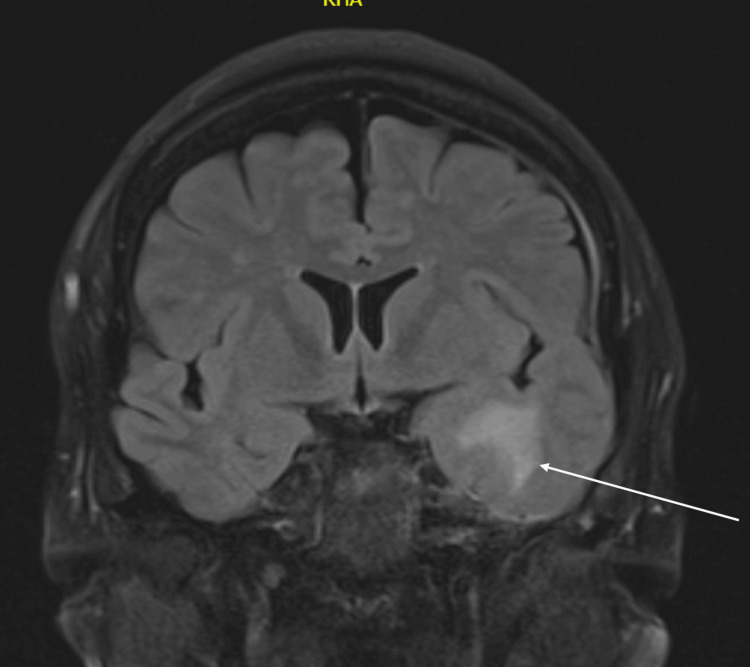
Fluid-attenuated inversion recovery (FLAIR) sequence magnetic resonance imaging (MRI). FLAIR sequence MRI showing gliotic changes in the left temporal lobe (white arrow). The annotated region corresponds to postoperative gliosis at the prior surgical site.

Laboratory investigations, including calcium, creatine kinase, thyroid function, vitamin B12, folate, and C-reactive protein, were within normal limits. A non-contrast computed tomography (CT) scan of the head showed no acute abnormalities. Magnetic resonance imaging (MRI) of the brain revealed chronic gliosis and encephalomalacia in the left temporal pole (Figure 1), corresponding to the previous surgical site, with no evidence of tumor recurrence, edema, or mass effect.

**Figure 2:**
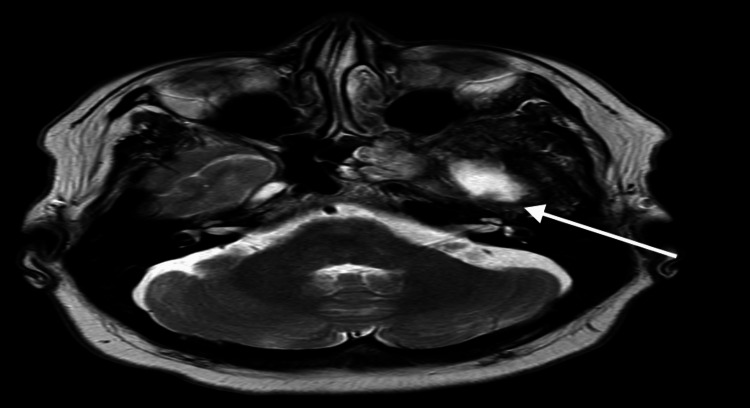
Diffusion-weighted imaging (DWI) MRI showing encephalomalacia in the left temporal lobe (white arrow). The annotated area highlights postoperative structural change without evidence of recurrence or mass effect.

Gliosis was also seen in MRI with diffusion-weighted imaging (DWI) (Figure 2).

Electroencephalography (EEG) showed a symmetric background rhythm with mild generalized slowing but no epileptiform activity or evidence of non-convulsive status epilepticus. Lumbar puncture demonstrated normal opening pressure, no pleocytosis, normal protein and glucose levels, and negative oligoclonal bands. Autoimmune and paraneoplastic panels were negative.

Given the subacute behavioral and cognitive decline following prior intracranial surgery, autoimmune and paraneoplastic encephalitis were initially considered, but clinical, imaging, and laboratory findings did not support these diagnoses. The patient was treated with a lorazepam challenge. This was titrated up to two milligrams four times daily, with only minimal improvement. Psychiatry, neurology, and neurosurgery jointly reviewed the case. Differential diagnoses included catatonia secondary to psychiatric illness, postoperative frontal-subcortical dysfunction, or delayed post-neurosurgical encephalopathy.

The skull base multidisciplinary team reviewed the neuroimaging and confirmed stable postoperative anatomy with no evidence of raised intracranial pressure or recurrence. ECT was deemed safe to proceed. Modified bilateral ECT was initiated under anesthetic supervision. The patient tolerated treatment well, with no neurological or cardiovascular complications. Gradual improvement was noted after the first few sessions, including resumption of oral intake, eye contact, and limited verbal responses. After six sessions, she showed progressive recovery, leading to continuation of ECT.

She began to improve gradually after six ECT sessions. After 24 sessions, she exhibited marked improvement in mobility, responsiveness, and affect. Cognitive testing using the Six-Item Cognitive Impairment Test (6CIT) improved from 23/30 to 12/30 within several weeks of ECT initiation [5]. These improvements were also seen in her Clinical Global Impression (CGI) and Montgomery-Åsberg Depression Rating Scale (MADRS) scores [6,7]. Key quantitative scores are summarized in Table 1.

**Table 1:** Quantitative clinical measures before and after ECT. The table summarizes key quantitative measures of catatonia, cognition, mood, and global functioning across the treatment course. BFCRS, Bush-Francis Catatonia Rating Scale; 6CIT, Six-Item Cognitive Impairment Test; MADRS, Montgomery-Åsberg Depression Rating Scale; CGI, Clinical Global Impression scale; ECT, electroconvulsive therapy

Measure	Pre-ECT	After six sessions	After 24 sessions	Interpretation
BFCRS (0-21)	8	4	1	Marked reduction in catatonic features
6CIT (0-28)	23	16	12	Cognitive improvement
MADRS (0-60)	38	22	12	Improvement in depressive symptoms
CGI (1-7)	6	4	2	Marked overall clinical improvement

Lithium was subsequently introduced to support mood stabilization and maintain functional recovery. At the time of writing, she remains under the care of a neurorehabilitation unit, receiving maintenance ECT every two weeks as part of a multidisciplinary program. The treatment continues to be well tolerated, with sustained gains in speech, spontaneity, affect, and social engagement, as well as gradual recovery of motor function, although she remains unable to walk independently.

## Discussion

Historically, intracranial pathology was regarded as a relative contraindication to ECT due to concerns regarding raised intracranial pressure and postoperative complications [3]. However, contemporary evidence has demonstrated that ECT can be safely administered in carefully selected patients with stable intracranial anatomy and no mass effect. Our case contributes to the growing body of literature supporting the safe and effective use of ECT in post-neurosurgical patients with catatonia.

Buday et al. [1] provide a comprehensive rationale for this shift, concluding that ECT can be safely administered to patients with intracranial tumors, particularly benign lesions such as meningiomas, when there is no mass effect or raised intracranial pressure. This is further supported by earlier case reports, including that of Gani and Parvez [2], which demonstrated the safe application of ECT in a patient with a history of meningioma resection [1]. The clinical course of our patient reinforces these findings, as multidisciplinary assessment confirmed stable postoperative anatomy, allowing for the safe and effective use of ECT in the management of treatment-resistant catatonia.

In our case, quantitative improvement across objective measures paralleled the patient’s clinical recovery. The BFCRS score decreased from 8 to 1, reflecting resolution of core catatonic features, including mutism, stupor, and posturing. Cognitive testing (6CIT) improved from 23 to 12, suggesting restoration of attention and executive function. Similarly, mood and global functioning improved, with MADRS scores reducing from 38 to 12 and CGI improving from 6 (*severely ill*) to 2 (*much improved*). These objective measures correspond with the patient’s observed re-emergence of speech, spontaneity, and affective responsiveness, demonstrating ECT’s multidomain therapeutic impact.

From a neurological perspective, the delayed onset of catatonia in our patient, nearly one year after surgery, raises the possibility of underlying network dysfunction contributing to the clinical presentation. This presentation shares features with the akinetic mutism syndrome described by Wangapakul et al. (2024) after removal of a bilateral parasagittal meningioma affecting the supplementary motor area (SMA) [8]. Our patient, who developed catatonia instead of immediate akinetic mutism post-surgery, exhibited similar psychomotor inhibition with intact consciousness. The delayed catatonia may reflect a variant of SMA dysfunction, potentially linked to postoperative changes in left frontal-basal ganglia-limbic circuits. Wangapakul et al. propose a continuum between acute SMA syndromes and later neurobehavioral disorders like catatonia, both characterised by network-level suppression of motor and speech initiation.

When compared with previous reports of post-neurosurgical catatonia, our patient’s course is noteworthy for the prolonged latency between surgery and symptom onset, the sustained functional recovery with maintenance ECT, and the absence of neurological complications. Similar cases reported by Gani and Parvez [2] and Swartz [9] describe shorter treatment courses and earlier response; however, our case demonstrates that extended ECT treatment (26 sessions) can produce outcomes comparable to or superior to those achieved with shorter courses when catatonia arises in structurally compromised but anatomically stable brain regions.

Furthermore, findings of Swartz [9] provide strong reassurance that ECT is a neurologically safe therapeutic option, even in patients with prior neurosurgical intervention or intracranial pathology. His review supports the absence of structural or cellular brain injury following ECT, aligning with other modern data that frame ECT as a controlled, physiologically benign neuromodulator treatment rather than a source of harm [10,11].

This case, therefore, highlights both clinical and conceptual lessons. Clinically, it demonstrates that ECT can be delivered safely and effectively in patients with a history of intracranial surgery when multidisciplinary evaluation confirms anatomical stability. Conceptually, it challenges the Cartesian distinction between *organic* and *functional* disorders. Catatonia, in this context, represents not an exclusively psychiatric or neurological entity, but a network-level dysfunction that bridges both domains. ECT, by restoring the integration of motor, cognitive, and affective functions, provides a means of bridging this divide.

## Conclusions

In summary, this report describes a patient who developed catatonia nearly one year after surgical resection of a meningioma. The patient had no prior psychiatric history and developed catatonia following a brain injury considered organic in origin by the treating team. The condition was unresponsive to pharmacological therapy but improved markedly following multidisciplinary review and initiation of ECT. To date, the patient has received approximately 26 ECT sessions and now demonstrates effective communication, independent transfers, and continued functional improvement.

This case supports the potential benefit and safety of ECT as an adjunctive treatment for catatonia in patients with a history of intracranial surgery, when anatomical stability is confirmed through multidisciplinary evaluation. Maintenance ECT may further enhance recovery when integrated into an overall neurorehabilitation approach, contributing to improved functional outcomes and quality of life.
